# Apoprotein L1 risk variants and kidney transplantation: a protocol for systematic review and meta-analysis

**DOI:** 10.11604/pamj.2022.42.77.33185

**Published:** 2022-05-27

**Authors:** Michael Abel Alao, Yemi Raheem Raji, Onyenunachi Asinobi, Adeolu Oladayo Akinboro, Samuel Osobuchi Ngene, Ibrahim Rasheed Olayinka, Ifeoma Okoye, Emmanuel Nna

**Affiliations:** 1 University College Hospital Ibadan, Ibadan, Oyo State, Nigeria,; 2 College of Medicine, University of Ibadan, Ibadan, Oyo State, Nigeria,; 3 Ladoke Akintola University of Technology Ogbomoso, Ogbomoso, Oyo State, Nigeria,; 4 Department of Epidemiology and Medical Biostatistics, University of Ibadan, Ibadan, Oyo State, Nigeria,; 5 Department of Paediatrics, Federal Medical Centre, Katsina State, Nigeria,; 6 Department of Radiation Medicine, University of Nigeria Teaching Hospital, Ituku Ozalla Enugu, Enugu State, Nigeria,; 7 The Molecular Pathology Institute, Independence Layout, Enugu, Enugu State, Nigeria

**Keywords:** Apoprotein L1 risk, renal transplantation, allograft, donor, recipient, modifiable factors

## Abstract

Graft survival after kidney transplantation may be influenced by both donors' and recipients' Apoprotein 1 (APOL1) risk variant status. There are several conflicting reports on screening, eligibility, and inclusion of APOL1 risk variant testing in the Kidney donor risk index. We developed a search strategy that included medical subject headings (MeSH), text words, and entry terms in order to search nine databases. The primary measurable outcome is the recipient's post-transplant graft survival time from APOL1 high-risk variant donors. The secondary outcomes are the proportion of APOL1 high-risk variants in end-stage kidney disease requiring a kidney transplant, the proportion in graft recipients and kidney donors; the effect of APOL1 high-risk variant on donor's kidney function post-kidney donation, recipient kidney allograft survival in APOL1 low and high-risk recipients. Confidence and comprehensive meta-analysis software will be used for the meta-analysis. Methodological, clinical, and statistical heterogeneity will be assessed. Publication bias will be visually assessed using the funnel plot. Results will be presented in forest plots with pooled survival time, standard error, and variance. Sub-group analysis will be performed using moderators such as sociodemographic characteristics, hypertension, HIV status, forms of rejection and other environmental factors. The primary outcome effect size is the standardized mean difference in survival time for APOL1 high risk variants in kidney transplants. The differences in kidney function between donors and recipients before and after transplantation would be examined. The suitability of donors with APOL1 high risk variants will be explored in terms of graft survival time, donor kidney function, and the aforementioned moderators.

## Introduction

Living donor kidney transplant accounts for 90% of kidney transplant in low middle-income countries, especially in sub-Saharan Africa [1]. Thus, the pools of donors are living members of the public sharing the same genetic risks and modifiable factors with the recipients [2,3]. The increasing selection criteria and eligibility for kidney transplantation may have improved the safety of kidney transplants as a procedure, the gaps, however, between organ demand and accessibility appear to be widened [4-6]. A recent review showed that a widening gap and disparity of living kidney donor and allograft recipients disproportionately impact the black population much more than the white counterpart [3,7]. Purnell *et al*. [8] suggested an ethnic group targeted education and orientation on the benefit of renal transplant for patients with end-stage kidney disease (ESKD), highlighting the potentially limited risk of donating a kidney. This will be the right direction to addressing organ shortage and closure of the ethnic-related chasm [8]. There are controversies on the criteria for the safety of organ donations in specific populations because of certain inherent peculiarities and delicate balance for organ availability [6]. Organ donation at the expense of donor´s safety could bear severe negative consequences on altruistic donors. Besides, a poor graft survival on the part of recipients renders this procedure worthless [8,9]. Therefore, it may be reasonable to fine tune screening criteria based on available evidence and weight of risk versus benefit to suit local resources. Some of the selection criteria may include screening for genetic factors such as APOL1 risk variant (genotype). Apoprotein 1 gene is a genetic risk marker linked to the development of non diabetic kidney disease in people of African ancestry. Its development in this sub population occurs as an adaptive epigenetic sequel to a natural selection process that followed infection with *Trypanosoma sp* [10,11].

The exposure to *Trypanosoma sp* infection could result in the development of 2 forms of high risk alleles associated with kidney diseases, referred to as APOL1 G1 and APOL1 G2 [10,11]. The APOL1 G1 and G2 variants protect from trypanosomiasis but predispose to the development of non diabetic kidney diseases often referred to as APOL1 associated nephropathy [10,11]. Up to 12-13% of African Americans have the two renal risk variants of the APOL1 gene that predispose to kidney disease [12]. Several studies have however shown that the presence of high-risk APOL1 alleles alone was associated with minimal risk for the development of APLO1 induced kidney disease among individuals of recent African ancestry [13-18]. The authors suggested the presence of a second hit or a modifying factor for the at-risk to develop kidney disease in the presence of high risk [18]. This mechanism has been postulated to be associated with the development of focal sclerosis and focal segmental glomerulosclerosis (FSGS) and rapid disease progression in patients with HIV-associated nephropathic [13-18]. Studies by Kofman *et al*. [19] and Zwang *et al*. [20] showed that transplants of a kidney from a living donor with two APOL1 renal risk variants (RRVs) could lead to de novo development of FSGS with early allograft failure in the recipient, and the development of ESKD in a previously healthy donor [19, 20]. The aforementioned report supports a cohort study involving 136 living donors of African ancestry who had two APOL1 RRVs after a median 12 years follow-up [21]. The report showed lower baseline estimated glomerular filtration rate(eGFR) in the donors, higher eGFR decline post-donation and an 11% rapid progression to ESKD within the follow-up period [21]. Other studies have shown that two RRVs of living kidney donor had a five-fold risk for Chronic kidney disease (CKD) [22]. However, in a review by Reeves-Daniel *et al*. [23], transplant of graft from African American to individual of Africa descent and Caucasian found no significant decline in recipient´s renal function [23]. They posited that the presence of APOL1 high-risk had no impact on graft survival in the recipient [23]. Similarly, Lee *et al*. [24] found no impact of APOL1 risk genotype of deceased donor kidney on five years´ graft survival of a similar ethic group, similar to findings from other studies [25,26]. Thus, data regarding the screening, eligibility and inclusion of APOL1 risk variant testing in the kidney donor risk index remain contemptuous. We aimed to produce a protocol for consistent and transparent systematic review and meta-analysis using available data on Apoprotein L1 risk variants, outcomes and modifiers of kidney transplantation.

**Objectives:** the main objective of this study is to determine the effects of APOL1 high-risk variants on the recipients kidney graft survival time, reported in standardized mean difference, and donor kidney function post kidney transplant.

## Methods

**Review questions:** 1) what is the pooled standardized mean difference (g) in graft survival time for APOL 1 low and high risk variants in donors and recipients?; 2) what is the pooled prevalence of APOL1 high-risk variant in ESKD, kidney transplant sub-population, kidney donors and graft recipients?; 3) what is the moderating effect of donors´ APOL1 high-risk variant on their kidney function post kidney donation?; 4) what is the moderating effect of donors´ APOL1 high-risk variant on recipients´ kidney allograft survival, in either low/high APOL1 risk types?; 5) what are the moderating effects of modifiable factors such race, age, gender, socio-economic status, forms of rejection, kidney function, HIV status, hypertension and other environmental factors on the recipient´s kidney allograft outcomes in donations from APOL1 high-risk variant donors?

### Study characteristics

**Study design:** this is a protocol for systematic review and meta-analysis of observational studies on APOL1 risk variants and kidney transplant. This protocol is designed to enable a reliable and accurate systematic review and meta-analysis on the impact of APOL1 risk variants on renal function of donor and recipient post-renal transplant. Using this protocol will enable determination of pooled effect sizes of graft survival (standardized mean difference g) from donors with APOL1 high-risk variants, donors´ kidney function post organ donation and to assess suitability of including APOL1 risk variant status in the Kidney donor risk index. There is no timeframe or restriction in selecting eligible studies using this protocol. In addition to study design, inclusion and exclusion criteria will be applied in selecting eligible studies.

**Inclusion criteria:** a) observational studies: cohort studies, case controls, cross-sectional studies, historic cohort studies; b) studies must report the primary outcome: post-transplant graft survival time from APOL1 high-risk donor. Studies must be retrievable in the English language.

**Exclusion criteria:** reviews, editorials, interventional studies, commentaries, methodological articles, letters to editors, case reports duplicates/replicates of studies. Studies not retrievable in the English language.

**Participants intervention comparison and outcomes (PICO):** the information for PICOs (participants, intervention, comparison, and outcomes) is provided below;

**Population:** kidney donors and recipients with APOL1 risk variants.

**Intervention:** kidney transplant

**Comparison:** graft survival in recipient with APOL1 low or high risk genotype

**Outcomes:** i) primary outcome: post-transplant graft survival from APOL1 high risk variant donor; ii)secondary outcomes: the proportion of APOL1 high-risk genotype in end stage kidney disease (ESKD) requiring kidney transplant, the proportion in graft recipients and kidney donors; iii)the summary effect sizes of APOL1 high-risk variants on donor's kidney function post kidney donation, recipient kidney allograft survival in APOL1 low and high-risk recipients; iv)the pooled summary effect size of the modifying factors on APOL1 high/low risk on individual kidney function of recipients and donor would be determined. This review will be reported in line with preferred reporting i for systematic reviews and meta-analyses (PRISMA 2015 statement) [27]. The protocol has the Prisma-P checklist attached as a supplementary material.

**Information sources:** the search will use sensitive topic-based strategies designed for each database. The search will be carried out in the following databases: Pubmed, Embase, Cinahl, research gate, Ajol, Google Scholar, Web of science, scopus and cohrane library. Only observational studies will be included.

**Search strategy:** the search strategy [((((("apolipoprotein L1"[MeSH terms'> ((((("apolipoprotein L1"[MeSH terms] or Apolipoprotein L1[word'>text word]) or ("Apolipoprotein-L1")) or ("APOL 1")) and (("kidney"['MeSH terms] or kidney [text word]) or (renal[all fields]))) and ((("Transplant*") or ("Dono*")) or ("Donat*"))) and ("risk"[MeSH terms] or risk[text word])] includes MESH terms, text words and entry terms. This was developed on PubMed and the same search terms will be used in the other databases with slight modifications.

### Data extraction and management

**Data extraction:** three main tools will be used for data extraction and management: a) covidence software; b) Microsoft Excel; c) comprehensive meta-analysis Software Version 3 Software. Six levels of data screening will be used for searched studies: i) level 1 would involve screening of identified studies for the study design. Only observational studies, retrievable in the English language would be selected; ii) level 2 will involve screening of studies in the titles and abstracts using entry terms, keywords and MeSH terms; iii) at level 3, studies will be further screened for content by reading the full text article using the same search strategy; iv) level 4 will involve snowballing of literature on references from included studies; v) level 5 will involve grey literature that report primary outcome and or secondary outcomes; vi) level 6, studies will be screened for outcomes, primary and secondary outcomes. Eight reviewers are involved in this study. A pair of reviewers will independently screen the identified articles for eligibility using covidence software [28]. The two reviewers will be blinded from each other using the screening tool. Conflict between the paired reviewers shall be resolved by a third reviewer who would will serve as a tie-breaker. The data of all screened studies will be deduplicated and exported to comprehensive meta-analysis software (CMA) version 3 for analysis [29]. Snowballing search of relevant studies through the review of the references of selected studies and grey literature will be performed manually.

**Selection process:** agreement between two independent and blinded reviewers who screened titles, abstracts, full texts of eligible observational studies, snowballed articles and grey literature will form the basis for selecting studies for inclusion for systematic review and meta-analysis. Where there are conflicts in decision, this will be resolved by a third reviewer. Authors of eligible studies with any missing data will be contacted via email and telephone.

**Data collection process:** extractable data items from selected studies will include the following: i) the last name of the first author and the year of publication; ii) sample size; iii) survival time for kidney transplant from APOL1 high risk variant donors; iv) number of kidney donors with APOL1 high-risk variants; v) changes in donor´s estimated glomerular filtration rate (eGFR)/Chronic kidney disease (CKD) stage before and after kidney donation; vi) number of kidney transplant recipients with APOL1 high-risk variants; vii) changes in recipient´s eGFR/CKD stage before and after kidney transplant; viii) the prevalence of the modifiable factors such as socio-demographics: race, age, gender and socio-economic status; hypertension, HIV status, forms of rejection and other environmental factors; ix) the adjusted risk ratio of the modifiable factors besides APOL1 high-risk. Data will be extracted into predefined forms created in Microsoft Excel.

**Data items/measurable outcomes:** the measurable outcomes are; i) standardized mean difference, g in survival time of kidney transplants involving APOL1 high risk variant donors; ii) proportion of kidney transplant recipients with APOL1 high-risk variants; iii) the proportion of kidney transplant donors with APOL1 high-risk variants; iv) changes in donor´s eGFR/CKD stage before and after kidney donation in relation to APOL1 risk variant status; v) changes in recipient´s eGFR/CKD stage before and after kidney transplant in relation to APOL1 risk variant status; iv) the proportion of the modifiable factors besides APOL1 high-risk; vi) the adjusted risk ratio of the modifiable factors besides APOL1 high-risk variants.

**Effect sizes:** the primary effect size is standardized mean difference g. Different primary indexes in individual studies of same design and report outcome will be converted to prevalence in the CMA Software. Categorical outcomes: race, gender and socio-economic status; hypertension, HIV status, forms of rejection and other environmental factors, will be used for sub-group analysis. Numerical outcomes such as age, kidney function variables will be used for meta-regression.

**Data synthesis:** the criteria for data synthesis are as follows: a) studies that passed the methodological quality assessment using the National Institute of Health (NIH) quality assessment tool for observational studies will be extracted. The results will be presented in tabular format; b) in addition to a narrative synthesis, the following will be included in the meta-analysis: i) studies with primary outcome will be included for systematic review. The primary outcome is standardized mean difference (g) in recipients´ graft survival time for donations involving APOL1 high risk variant donors; ii) studies with both primary and secondary outcomes will be included for meta-analysis.

**Quantitative analysis:** criteria for quantitative data synthesis studies that are used in narrative synthesis, which also report both primary and secondary outcomes will be included for meta-analysis. Data items will be used to generate standardized mean difference, standard error, variance and 95% confidence interval. Heterogeneity will be assessed using the Q statistics and its p-value, tau^2^and the Higgins I^2^, I^2^values of less than 40% will be considered low heterogeneity while values > 40 but < 75% will be considered moderate and values > 75% are high. A random-effect model will be used for computation in this study. A sensitivity analysis will be performed to check for outlying studies and their effects on standardized mean difference, pooled mean and standard deviation (SD) for changes in eGFR. Publication bias in the selection of studies will be visually assessed on the funnel plot (trim and fill method) and tested for asymmetry. Other statistical tests such as Egger´s regression intercept, Begg and Mazumdar's rank correlation and Orwin´s fail-safe N will be used where appropriate. For each included study, the primary outcome which is the standardized mean difference (g) in recipient´s graft survival time for donations from APOL1 high risk variant donors will be used in calculating the pooled g value, standard error, variance and 95% continuous integration (CI) of variance. This will be reported in forest plots. Sub-group analysis will be performed using categorical data such as race, gender, socio-economic status, forms of rejection, hypertension, HIV status and any modifiable factor. All subgroup analysis will be presented in forest plots. Meta-regression will be performed on quantitative explanatory variables such as changes in eGFR in donor and recipient before and after transplant, age and proteinuria (if quantified). Quantitative analysis will be done using the Comprehensive meta-analysis (CMA) software version 3 (Biostat, USA) [29].

**Risk of bias:** the risk of bias will be assessed for each included study using the National Institute of Health (NIH) Quality assessment tool for observational cohort and cross-sectional studies. The NIH quality assessment tool has 14 questions. Scores above 7 show good quality study with less bias. This will be cross-checked with the Cochrane tool of risk of bias assessment. Studies with extreme bias will be subjected to sensitivity testing using the include/exclude function in the CMA software.

**Assessment of meta-bias:** meta-bias will be assessed as follows: i) method of testing/reporting of APOL1 high-risk variants in kidney transplant recipients and donors. This will be done at outcome level; ii) reporting of study: studies that were reported in different units but similar in outcome and design will be converted based on individual case evaluation. This will be evaluated for individual studies by assessing the unit of reporting of studies, for example, whether mean SD, prevalence with confidence intervals or incidence or proportion are reported. This is done at outcome level; iii) heterogeneity will be assessed at the study level using the Q statistics and its p-value, tau^2^and the Higgins; iv) bias will be assessed at the study level using the funnel plot (trim and fill method) and test for asymmetry; v) sensitivity analysis will assessed at the study level using include and exclude function in the CMA software.

**Presentation and reporting of results:** the study selection process will be summarized in a Prisma flow chart according to the Prisma 2015 statement and Prisma-P checklist. A table of the search strategy in various databases showing text words, MeSH and entry terms will be presented. List of included studies will be summarized in a table. Quantitative data such as standardized mean difference and pooled g, standard error and 95% continuous integration (CI), p values, relative weights assigned to studies and heterogeneity tests will be included in the forest plots. A table of quality scores and risk of bias of each eligible study will be presented. Forest plots to show sub-group analysis will be included. Meta-regression and sensitivity analysis will be shown in figures and tables respectively.

**Trial registration number:** this protocol is registered in Prospero, with registration number CRD42021230358.

## Discussion

The effect size for primary outcome is standardized mean difference in survival time for APOL1 high risk variants in kidney transplants. The changes in kidney function of donors and recipient pre- and post-transplantation would be examined. The suitability of donors who have APOL1 high risk variants will be explored in relation to graft survival, donors´ kidney function and moderating effects of sociodemographic and environmental determinants. The discussion will assess the possible value of including APOL1 risk variant status of donors and recipients in Kidney donor risk index. Further discussion will include the effects of moderating and modifiable factors in graft survival time.

**Ethics and dissemination:** the study will use published data, thus, no ethical approval is required. The final report of this study will be published in a peer-reviewed scientific journal and made available to medical experts in the field of kidney transplantation.

**Funding:** the study is funded by the authors.
